# Isolation of a euryhaline microalgal strain, *Tetraselmis* sp. CTP4, as a robust feedstock for biodiesel production

**DOI:** 10.1038/srep35663

**Published:** 2016-10-21

**Authors:** Hugo Pereira, Katkam N. Gangadhar, Peter S. C. Schulze, Tamára Santos, Carolina Bruno de Sousa, Lisa M. Schueler, Luísa Custódio, F. Xavier Malcata, Luísa Gouveia, João C. S. Varela, Luísa Barreira

**Affiliations:** 1 CCMAR-Centre of Marine Sciences, University of Algarve, Campus de Gambelas, 8005-139 Faro, Portugal; 2 LEPABE-Department of Chemical Engineering, Faculty of Engineering, University of Porto, Porto, Portugal; 3 Department of Chemical Engineering, Rua Dr Roberto Frias, P-4200-465 Porto, Portugal; 4 LNEG-Laboratório Nacional de Energia e Geologia, I.P./Bioenergy Unit, Estrada do Paço do Lumiar 22, 1649-038 Lisboa, Portugal

## Abstract

Bioprospecting for novel microalgal strains is key to improving the feasibility of microalgae-derived biodiesel production. *Tetraselmis* sp. CTP4 (Chlorophyta, Chlorodendrophyceae) was isolated using fluorescence activated cell sorting (FACS) in order to screen novel lipid-rich microalgae. CTP4 is a robust, euryhaline strain able to grow in seawater growth medium as well as in non-sterile urban wastewater. Because of its large cell size (9–22 μm), CTP4 settles down after a six-hour sedimentation step. This leads to a medium removal efficiency of 80%, allowing a significant decrease of biomass dewatering costs. Using a two-stage system, a 3-fold increase in lipid content (up to 33% of DW) and a 2-fold enhancement in lipid productivity (up to 52.1 mg L^−1^ d^−1^) were observed upon exposure to nutrient depletion for 7 days. The biodiesel synthesized from the lipids of CTP4 contained high levels of oleic acid (25.67% of total fatty acids content) and minor amounts of polyunsaturated fatty acids with ≥4 double bonds (<1%). As a result, this biofuel complies with most of the European (EN14214) and American (ASTM D6751) specifications, which commonly used microalgal feedstocks are usually unable to meet. In conclusion, *Tetraselmis* sp. CTP4 displays promising features as feedstock with lower downstream processing costs for biomass dewatering and biodiesel refining.

Microalgal biomass has drawn increasing attention for different biotechnological applications over the last few years, as shown by the significant developments in terms of funding allocated for microalgal research. The establishment of several start-up companies and commercial products from microalgae (e.g. DSM Life’s^®^ and Qualitas Health^®^ nutraceuticals as well as Encapso^®^ drilling oil) have confirmed that these microorganisms can be important feedstocks in different markets. Nevertheless, recent achievements observed in the field of microalgal biotechnology were mainly due to research and innovation efforts towards the development of microalgae-based biofuels^1^. Although the technology to produce biofuels (e.g. biodiesel) from microalgal biomass has been successfully demonstrated, several techno-economical reports concluded that the biofuels obtained from microalgal feedstocks are still unable to compete with fossil fuels^2^,^3^,^4^. To overcome the current constraints for the commercialization of microalgae-based biofuels, the optimization of the whole production pipeline is required. An important first step is strain selection, which has to take into account later steps, such as its robustness, easy and low cost downstream processing and the effective development of a biorefinery.

During the last decades, aquaculture has been the main market for microalgal biomass, particularly for rearing bivalves and enhancing the nutrition of live prey^5^,^6^. Since unsaturated lipids are essential metabolites for the proper development of fish larvae and bivalve growth^7^, microalgal strains with high contents of polyunsaturated fatty acids (PUFA) have been selected and commercialized. However, microalgal feedstocks with high amounts of PUFA are not suitable for biodiesel production, because the large number of double bonds present in these fatty acids decrease the oxidation stability of the end product^8^,^9^,^10^. In fact, both European (EN14214) and American (ASTM D6751) specifications impose strict limits concerning the presence of PUFA in biodiesel. Therefore, screening for strains containing high lipid content with low levels of unsaturated fatty acids is crucial to enhance the productivity and quality of the feedstock used for biodiesel production^10^,^11^,^12^ and decreases the costs of biodiesel refining^11^.

Flow cytometry coupled to fluorescence activated cell sorting (FACS) is a powerful high-throughput technique for bioprospecting microalgae present in environmental samples, because this technology enables the screening of thousands of cells in a short period of time for a specific purpose (e.g. high lipid contents). This can be accomplished by acquiring different signals, such as complexity and relative cell size coupled with the autofluorescence of photosynthetic pigments and the fluorescence of solvatochromic dyes^13^,^14^,^15^. Depending on the final product, bioprospection using FACS enables the isolation of strains with a desired biochemical profile through different selection approaches (e.g. dyes that only emit fluorescence in the presence of lipids), narrowing down the number of strains that have indeed a high potential for a given biotechnological application^16^,^17^. Concomitantly, selection procedures must also contemplate the growth performance and robustness of a given strain, as both features are crucial for the up-scaling and effective production in large-scale systems^18^.

This work aimed to characterize and evaluate the potential of a novel euryhaline microalga isolated from a salt marsh near a wastewater stream in the south of Portugal as a feedstock for biodiesel production. *Tetraselmis* sp. CTP4 was selected from 96 isolates as a result of a screening effort using FACS to bioprospect for novel microalgal strains with biotechnological potential.

## Results

### Microalgae isolation and identification

**Figure 1:**
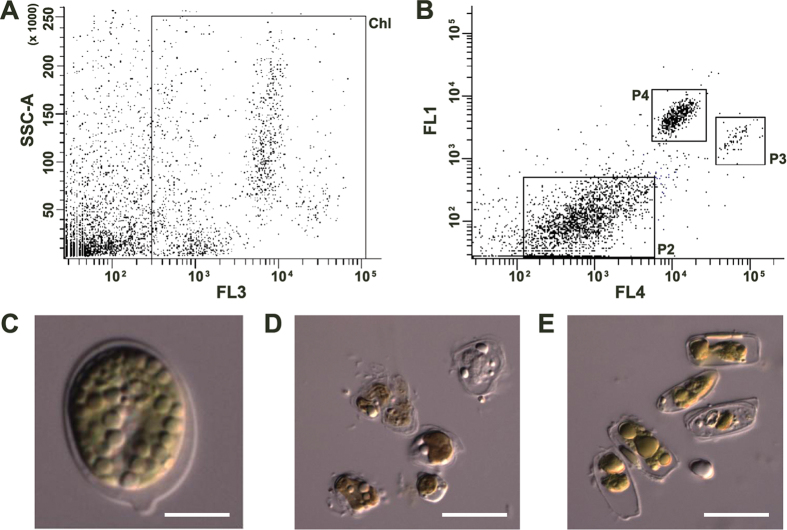
Cell sorting procedure used to isolate CTP4 strain by means of fluorescence activated cell sorting (**A,B**), and different clusters sorted directly onto microscope slides observed with differential interference contrast (**C–E**). The first sorting trait (Chl gate) was applied through the combination of inner cell complexity, SSC-A, with chlorophyll autofluorescence, FL3 (**A**). The final gates used to isolate the CTP4 strain combined BODIPY fluorescence (FL1) with the signal of the allophycocyanin autofluorescence channel (FL4). Strains isolated from clusters P4 (**C**), P2 (**D**) and P3 (**E**). The first strain was named as CTP4 (**C**), whereas the second corresponded to an unidentified strain with apparent cell disruption (**D**) and the third strain was an unidentified diatom (**E**). Scale bar = 5 μm.

*Tetraselmis* sp. CTP4 was isolated by FACS using the sorting procedure shown in Fig. 1. Figure 1A presents the two-dimensional plot combining the SSC (side angle light scatter) and FL3 (fluorescence emission at 695 nm) signals, relating the relative inner cell complexity with chlorophyll autofluorescence, respectively. Through the combination of these signals, the first sorting trait was established in order to differentiate non-photosynthetic from photosynthetic cells; in this way, further analyses focusing only on photosynthetic cells were carried out^17^. Figure 1B shows the combination of the allophycocyanin autofluorescence signal (FL4) and the emission of BODIPY 505/515 (FL1), a lipid-staining solvatochromic dye. With this combination of signals, three clusters of cells representing three different microalgal species could be clearly distinguished. Two (P3 and P4) of the three clusters displayed higher values of BODIPY fluorescence. The gates used in the sorting procedure were effective for the isolation of cells belonging to three different species as verified by microscopy upon sorting directly onto microscope slides (Fig. 1C–E). The isolates were entitled CTP3, CTP4 and CTP5, according to the numbering of the clusters obtained in the cytometer. Interestingly, among all isolates, CTP4 presented the most dense cluster of events as compared with other microalgae found in the environmental sample (Fig. 1B). This was an important first indicator that CTP4 was able to compete with other microalgae that were co-cultivated during the pre-enrichment step of the isolation process. Moreover, this microalga also showed the highest levels of BODIPY fluorescence (FL1), which strongly suggested that this microalga contained significant amounts of lipids. As a result, CTP4 was selected for further study.

**Figure 2:**
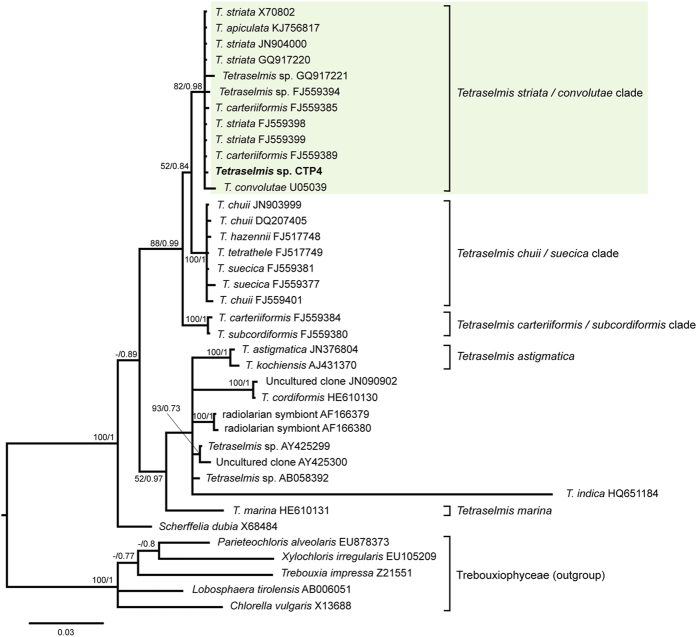
Bayesian inference tree of species of the Chlorodendrophyceae class inferred using 18S rDNA sequences. Maximum-likelihood bootstrap values (>50) and Bayesian inference posterior probabilities (>0.70) are indicated at the branches, respectively. The CTP4 strain, isolated in this study, clustered with the *striata*/*convolutae* clade of the *Tetraselmis* genus.

Chlorodendrophyceae 18S rDNA sequences were analysed by Bayesian (BI) and Maximum Likelihood (ML) inference and the consensus tree is shown in Fig. 2 using equivalent sequences from Trebouxiophyceae algae as the outgroup. Topology of the BI and ML consensus trees indicates that *Tetraselmis* sp. CPT4, isolated in this study, belongs to the *T. striata*/*convolutae* clade with a posterior probability of 0.98 and a bootstrap value of 82%, respectively (Fig. 2).

### Culture robustness and dewatering

**Figure 3:**
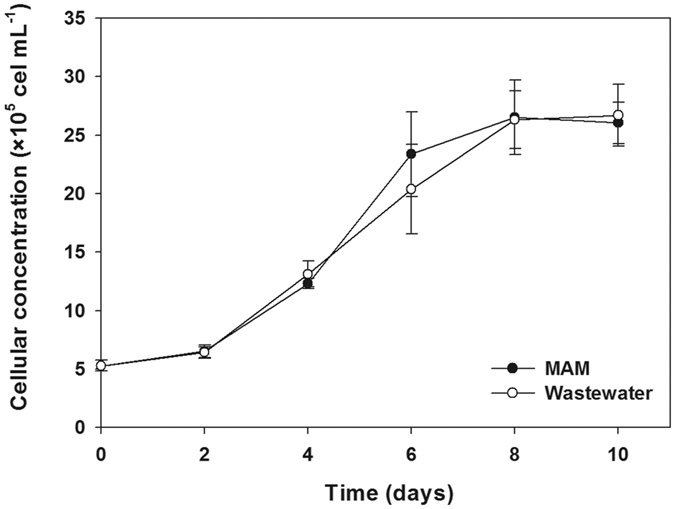
Growth curves of *Tetraselmis* sp. CTP4 cultures grown in standard conditions and a wastewater effluent. Cultures were grown in Modified Algal Medium (MAM, salinity ≈ 3.6%) and non-sterile urban effluent (salinity ≈ 0.5%) for 10 days with a starting inoculum of 5 × 10^5^ cells mL^−1^.

To further characterise the novel strain, *Tetraselmis* sp. CTP4 was cultivated in seawater-based Modified Algal Medium (MAM) and in a non-sterile, non-nitrified wastewater effluent (Fig. 3). Cultures were grown until a cell concentration (CC) of about 2.7 × 10^6^ cells mL^−1^ was reached. Both batch cultures displayed similar growth curves, reaching stationary phase at day 8. Cultures were monitored daily by bright field microscopy and flow cytometry. Although bacterial and microalgal contaminants were frequently observed in the non-sterile wastewater culture, *Tetraselmis* sp. CTP4 remained the dominant specie throughout.

**Figure 4:**
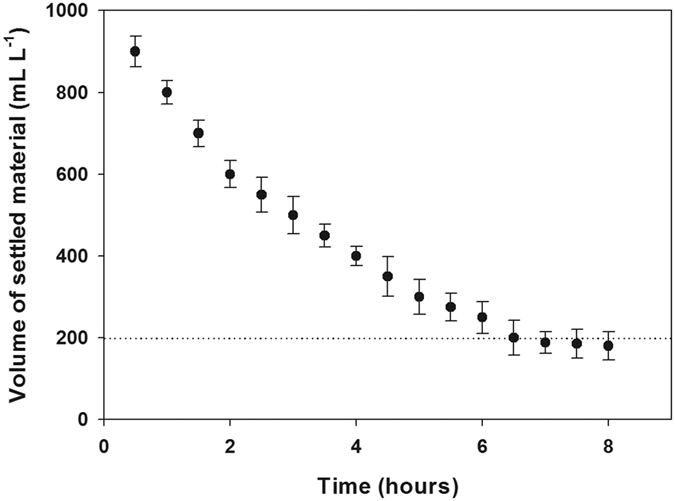
Volume of settled material of *Tetraselmis* sp. CTP4 cultures in Imhoff cones (*n* = 3).

The natural sedimentation of cultures, presented as volume of settled culture per litre of culture, was investigated during the course of 8 hours using Imhoff cones (Fig. 4). The settling of the cultures revealed a decreasing logarithmic curve, converging towards 18% of the initial volume over 6 hours.

### Biomass growth and lipid induction

**Figure 5:**
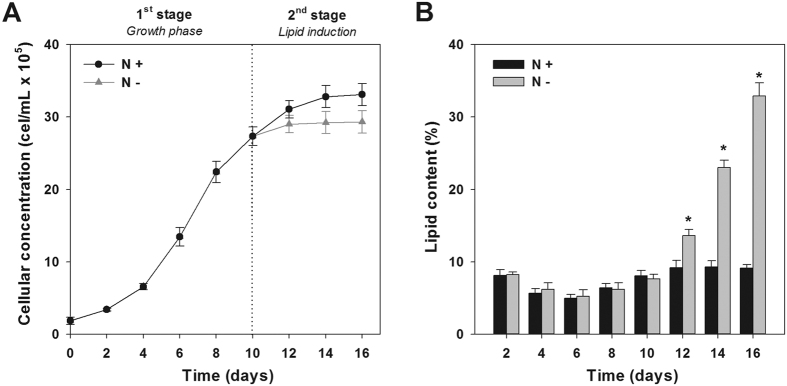
Cultures grown in a two-stage system under nutrient repletion (N+) and nutrient depletion (N−) conditions. The growth curves (**A**) and the corresponding mean lipid content (**B**) of cultures exposed to both culture conditions is shown (*n* = 3). Dashed line shows the addition of nutrients to the N+ cultures (**A**).

**Figure 6:**
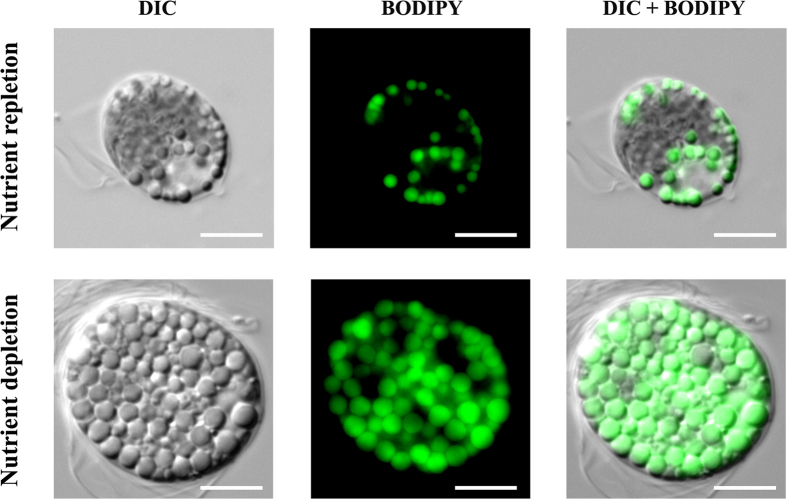
BODIPY 505/515 staining of *Tetraselmis* sp. CTP4 cells. Images were acquired using differential interference contrast (DIC) and BODIPY fluorescence, showing the lipid bodies in cells grown under nutrient repletion (N+) and nutrient depletion (N−) media. Scale bar = 5 μm.

The growth curves of cultures grown under nutrient repletion (N+) and nutrient starvation (N−) in a two-stage growth system are shown in Fig. 5A. Both cultures grew exponentially until the end of the 1^st^ stage (day 10), reaching a CC of 2.9 × 10^6^ cells mL^−1^. The specific growth rate (μ) during this stage was similar in both culture conditions (0.29–0.31 d^−1^). In the 2^nd^ stage, N+ cultures continued to grow, although at a slower rate, reaching a final concentration of 3.3 × 10^6^ cells mL^−1^, whereas the N− cultures plateaued at approximately 3.0 × 10^6^ cells mL^−1^. At the beginning of the 1^st^ stage the lipid content of both cultures was approximately 10% of DW (Fig. 5B). During the exponential phase (between day 4 and 8), a decrease in the lipid content (5–8% of DW) was observed. During the 2^nd^ stage (lipid accumulation stage), cultures supplemented with nutrients (N+) maintained the same lipid content, displaying a final lipid content of 10% of DW. However, the N− cultures reached a significantly higher lipid content: approximately 33% of DW. These results were confirmed by staining with BODIPY 505/515; microalgae grown under nutrient starvation contained a significantly higher amount of lipid bodies that stained positively for the solvatochromic dye (Fig. 6). The biomass and lipid productivities obtained in the present study and previous reports with other *Tetraselmis* strains are presented in Table 1. The N+ cultures displayed higher biomass productivity compared to those in the N− treatment, yielding 0.29 and 0.25 g L^−1^ d^−1^, respectively. On the other hand, the final lipid productivity doubled under the N− conditions (52.1 mg L^−1^ d^−1^) when compared to N+ cultures (24.5 mg L^−1^ d^−1^).

### Biochemical profile and properties of CTP4 biodiesel

Upon synthesis of biodiesel derived from the lipids extracted from CTP4 cells, the fatty acid methyl esters (FAME) profile of the biofuel was determined (Table 2). Palmitic (C16:0), oleic (C18:1) and linoleic (C18:2) acids were the major FAME detected, representing approximately 75% of the total fatty acids (TFA) in the biodiesel mixture. Other FAME also found at relatively high amounts were palmitoleic (C16:1) and hexadecatrienoic (C16:3) acids (10% of TFA), whereas only minor levels of hexadecadienoic (C16:2), linolenic (C18:3) and eicosapentaenoic (C20:5n-3, EPA) acids were detected. The properties of the synthesised biodiesel were then determined (Table 3) and compared to the limits established by the international biodiesel specifications (EN14214 and ASTM D6751). All FAME related properties, namely total FAME, cetane number (CN), iodine value (IV), cold filter plugging point (CFPP), linolenic acid and PUFA ≥4 double bonds (db) contents, were within or close to the values established in the EN14214 specification. The measured density, kinematic viscosity and oxidation stability were 0.85 Kg L^−1^, 3.64 mm^2^ s^−1^ and 4.74 h, respectively. Produced biodiesel was devoid of glycerol (total and free) and acylglycerols (mono-, di- and triacylglycerols). The levels of group I (Na + K) and group II (Ca + Mg) metals were 0.45 and 0.05 mg Kg^−1^, respectively. Hence, both values were below the maximum limits specified by both standards. The phosphorous content was, however, higher than the specified limits.

## Discussion

Microalgae intended for large-scale production need to be robust and present high growth rates in order to withstand wide environmental conditions and outgrow competitors and predators. In this sense, the enrichment step carried out before the FACS isolation step promoted the isolation of strains able to outcompete other cells also found in environmental samples. This step is thus crucial for the selection of robust microalgae that are able to become dominant even under challenging conditions and in the presence of contaminants^17^,^19^. Strains of interest for biodiesel production should develop clusters with a higher number of events during FACS isolation, combined with a higher lipid-BODIPY signal. From an initial pool of 96 isolates obtained by authors’ FACS-based methodology, *Tetraselmis* sp. CTP4 was selected as a promising biodiesel feedstock due to the combination of dominance over contaminants and lipid content. Strain identity was confirmed by phylogenetic analysis showing that *Tetraselmis* sp. CTP4 belongs to the *striata*/*convolutae* clade, in accordance with data reported by Arora *et al*.^20^.

The robustness of this euryhaline microalga was evaluated by growing cultures in non-sterile urban wastewater. *Tetraselmis* sp. CTP4 displayed similar growth curves in both the control MAM and the wastewater and, most importantly, it dominated over the microorganisms naturally present in the wastewater. Strains isolated in areas of wastewater discharges usually show high tolerance to oxidative stress and are often well suited for wastewater treatment^21^. Moreover, recent trials have shown that *Tetraselmis* sp. CTP4 can grow at salinities ranging from ~1 to 100‰ (data not shown). Halotolerant strains display a key advantage for large-scale production, since the manipulation of the salinity (high vs. low salt shifts) in the culture medium can manage and contain possible contaminants, without affecting significantly the biomass productivity of the cultures. This feature is also a key feature to recycle the marine culture medium after dewatering, upon which wide variations of salinity can occur, as recently reported by Fon-Sing *et al*.^22^ in pilot scale open raceways used to grow a euryhaline *Tetraselmis* strain.

Harvesting/dewatering of cultures is a main constraint in the whole microalgal production pipeline, due to the high-energy demands associated with biomass recovery from massive amounts of water^23^,^24^. In this sense, the ability of *Tetraselmis* sp. CTP4 to settle down naturally is another crucial advantage, as it allows the removal of 80% of the culture medium after a 6-h sedimentation step without the addition of flocculants or the use of a pre-concentration procedure. Through this approach only 20% of the culture volume needs to be harvested using common methods (e.g. centrifugation and filtration), having thus the potential of decreasing significantly the costs of biomass dewatering.

To compare the lipid production of cultures, cells were cultured under nutrient repletion (N+) and depletion (N−) using a two-stage growth system. Results showed that cells grown under nutrient depletion yielded a 3-fold increase in the total lipid content and significantly higher cell size (15–22 μm) than the N+ treatment (9–12 μm). Lipid accumulation was only triggered upon nutrient starvation, which might affect amino acid levels needed for protein synthesis, providing a higher number of carbon skeletons that will be available to triacylglycerol biosynthesis^25^,^26^. The two-stage approach used for inducing lipid accumulation in *Tetraselmis* sp. CTP4 effectively improved the lipid productivity of this microalgae, leading to a 2-fold increase in cultures exposed to nutrient depletion. The results of this two-stage system are in accordance with Gouveia *et al*.^27^ and Campenni’ *et al*.^28^. However, they do not match the results recently published by Kim *et al*.^29^, where N+ cultures displayed higher lipid productivity. Although a strain-dependent response cannot be excluded, such difference may be explained by the short induction period of the 2^nd^ stage (36 hours) used by these authors, which probably did not enable an effective lipid induction in the N− cultures. In the present work, the two-stage approach only promoted lipid accumulation in *Tetraselmis* sp. CTP4 after 48 hours of nutrient starvation.

The overall biomass and lipid productivities established in the present work for *Tetraselmis* sp. CTP4 matched those from previous reports on other *Tetraselmis* strains (Table 1). Moreover, chlorophytes and particularly those of the *Tetraselmis* genus have already been shown to be able to grow in outdoor systems, and can be promising feedstocks for the production of microalgae-based biofuels^30^,^31^,^32^. The latter conclusion is confirmed by the fact that biodiesel synthesised from wet biomass of *Tetraselmis* sp. CTP4 displayed values within or close to the limits defined by the EN14214 and ASTM D6751 specifications (Table 3). From all properties investigated, the phosphorus content was the only parameter clearly outside the limits described in both specifications. However, this result was expected since, generally, microalgae oils present a significant amount of phospholipids^33^,^34^ that are co-extracted with the triacylglycerols. Therefore, removal of phospholipids (e.g. degumming) from the microalgae oil is essential in order to reduce the content of phosphorus in microalgal biodiesel and fulfil the limits of both specifications^34^,^35^.

Most microalgae strains fail to address the properties related with the saturation of the lipid profile, namely, the content of linolenic acid, PUFA ≥4 db, IV, CN, and most importantly, the oxidation stability^8^,^10^,^36^. In fact, the oxidative stability of the produced biodiesel (4.74 h) is to the authors’ knowledge the highest value reported for B100 microalgae-based biodiesel, except for that of *Scenedesmus* sp. (5.42 h)^37^. Perrier *et al*.^11^ and Chen *et al*.^37^,^38^ produced B100 biodiesel from *Chlorella protothecoides, Nannochloropsis* sp. and a dinoflagellate with induction periods of 4.52, 0.8–1.93 and 1.02 h, respectively. The oxidative stability of CTP4 biodiesel was also significantly higher than the values previously reported for the biodiesel produced from other vegetable sources, such as soybean (3.9 h), palm (3.52 h), rice bran (1.7 h) and sunflower (0.4 h) oils^39^,^40^,^41^,^42^. Such oxidative stability is probably related with the FAME profile of CTP4 and consequently of the produced biodiesel that revealed only trace values of long-chain PUFA, and high contents of palmitic, oleic and linoleic acids, accounting for nearly 75% of the TFA. The FAME profile of the biodiesel produced from CTP4 presents a lower degree of unsaturation than those of other *Tetraselmis* strains previously published in the literature^10^,^31^,^43^,^44^. The same is observed when the lipid profile of CTP4 is compared with that of most common commercial strains of microalgae, such as *Nannochloropsis oculata* and *Phaeodactylum tricornutum*^10^. The low unsaturation degree of *Tetraselmis* sp. CTP4 is a crucial advantage for biodiesel production, as recently highlighted in several reports^8^,^10^,^11^,^12^.

In conclusion, *Tetraselmis* sp. CTP4 displays several promising features as a biodiesel feedstock, including robustness, high biomass and lipid productivities, and potential for reduced downstream costs related with biodiesel refining and biomass dewatering.

## Methods

### Microalgae isolation and culture scale-up

*Tetraselmis* sp. CTP4 was isolated near a wastewater treatment plant in Ria Formosa, a coastal lagoon located in the south of Portugal (Algarve), by a microplate-based high throughput screening procedure described in Pereira *et al*.^17^. Briefly, water samples were supplemented with concentrated MAM^17^ and left exposed to indirect sunlight for approximately 1–2 weeks. Afterwards, aliquots were taken and stained with BODIPY 505/515 (4,4-difluoro-1, 3, 5, 7-tetramethyl-4-bora-3a,4a-diaza-*s*-indacene; Life Technologies Europe BV, Porto, Portugal) as described in Cooper *et al*.^45^ to prepare for flow cytometry. Stained samples were acquired in a Becton Dickinson FACS Aria II (BD Biosciences, Erembodegem, Belgium) equipped with a blue and red laser (488 and 633 nm, respectively) and FACSDiva (version 6.1.3) software. Four channels were used to record the fluorescence signal, namely FL1, FL2, FL3 and FL4 centred at 530/30, 585/42, 695/40 and 660/20 nm, respectively, after excitation with the blue (FL1-FL3) or red (FL4) laser. Cells emitting higher levels of fluorescence due to chlorophyll pigments and lipids stained with BODIPY were sorted directly onto 96-well microplates containing 250 μL of solid (agar) MAM and onto microscope slides. Colonies growing in the wells of the microplates were transferred to Petri dishes containing agar supplemented with MAM. The biomass growing on the Petri dishes was scrapped and transferred to 100-mL Erlenmeyer flasks with sterilized seawater and MAM and later transferred to 1-L photobioreactors with aeration.

### Microscopy

Microscopic images were acquired in a Zeiss AXIOMAGER Z2 microscope, with a coollSNApHQ2 camera and AxioVision software version 4.8 (Carl Zeiss MicroImaging GmbH, Göttingen, Germany), using the 100 × lens. Brightfield microscopy was carried out using differential interference contrast (DIC), while Zeiss 38 He filter set (Carl Zeiss MicroImaging GmbH, Gõttingen, Germany) for fluorescein isothiocyanate (FITC) was used to acquire the fluorescence images. Samples used for fluorescence microscopy were stained with BODIPY 505/515 as described for the flow cytometry analysis. Images were treated using Image J software (Research Service Branch, NIH, Bethesda, MD).

### Taxonomic identification

This strain was identified by means of 18S rDNA sequencing. DNA extraction was performed with the EZNA DNA plant extraction kit (Omega Bio-Tek, Norcross, GA) according to the manufacturer’s guidelines. The obtained DNA was amplified by PCR with the primers 18SUnivFor (5′-ACCTGGTTGATCCTGCCAGT-3′) and 18SUnivRev (5′-TCAGCCTTGCGACCATAC-3′) as described in Pereira *et al*.^17^,^19^ and sequenced at an in-house DNA sequencing facility equipped with an Applied Biosystems 3130XL DNA sequencer (Life Technologies BV, Porto, Portugal). The obtained sequence was deposited in GenBank with the accession number KX278369, and compared with the GenBank database using BLASTn (https://blast.ncbi.nlm.nih.gov). The sequences were aligned and visually inspected using CLC Sequence Viewer (v. 7.6.1, Quiagen) and curated with Gblocks v. 0.91b software^46^. Curation was performed allowing gap positions within the final blocks and a maximum of 8 contiguous nonconserved positions and a minimum block length of 5 nucleotides. Phylogenetic analysis was performed using Maximum-likelihood (ML) and Bayesian inference (BI). The substitution models that best fit the data set were selected using MrModeltest2 v.2.3^47^ and PAUP* v.4.0b10^48^ applying the Akaike information criterion (AIC; Akaike 1974). ML analysis was performed using RaxML v. 7.0.4^49^, assuming a GTR + I + G substitution model with 400 bootstrap replicates. Posterior probabilities were determined by Markov Chain Monte Carlo (MCMC) sampling in MrBayes v. 3.1.2^50^,^51^. MrBayes analyses were also conducted using the model GTR + I + G with 6 chains for 10,000,000 MCMC generations, sampling every 1,000th generation and using the default for all the other settings. The MCMC runs convergence and burn-in were determined through the analysis of the generations vs. log probability plot using the trace analysis tool TRACER v1.6^52^. The final tree was drawn with FigTree v.1.3.1^53^.

### Microalgae growth

All experiments were performed in a specialized growth chamber (Aralab Fitoclima S 600 PL clima plus 400), at 20 ± 0.5 °C, under continuous lighting (100 μmol m^−2^ s^−1^). Cultures were grown using 100 mL glass reactors, aerated continuously with filtered (0.2 μm) compressed air. Standard culture medium was seawater (salinity = 36‰) supplemented with MAM. For the wastewater experiment, non-nitrified sewage effluent was supplied by Quinta do Lago wastewater treatment plant (WWTP, Quinta do Lago, Algarve, Portugal).

### Lipid induction

Lipid induction assays were carried out using a two-stage growth system. Cultures were grown until day 10, under controlled conditions, as described in the previous section to allow the optimal growth of cultures reaching a high cell concentration (1^st^ stage). At this stage, the nitrate content of the growth medium was completely depleted as determined by spectrophotometric methods described in APHA^54^. At day 10, the produced inoculum was either supplemented with concentrated MAM (N+, nutrient replete) or was left without nutrients (N−, nutrient depleted) to promote lipid induction (2^nd^ stage). All the experiments were carried out in triplicate and average values are reported. Results were statistically analysed using SPSS (release 15.0, SPSS Inc., Chicago, IL) software, using analysis of variance (one-way ANOVA) and Tukey HSD post-hoc test with a confidence interval of 95%.

### Growth evaluation and chemical analysis of cultures

#### Determination of algal growth

Microalgal biomass concentration was determined by measuring the optical density of the cell culture in a 96-well plate spectrophotometer (Biotek Synergy 4) at 750 nm. CC was measured using a Neubauer counting chamber according to the manufacturer indications and through flow cytometry using CountBright™ absolute counting beads. For biomass concentration, expressed in a dry weight (DW) basis, 10 mL of algal suspension was filtered through a 0.45 μm cellulose acetate filter, washed with ammonium formate (37 g L^−1^) and dried in an oven with forced air circulation at 60 °C until constant weight.

#### Total lipid determination

Total lipid content was determined following the Bligh & Dyer method^55^ with a few modifications as described in Pereira *et al*.^17^. Briefly, biomass was extracted with a mixture of chloroform, methanol and water (2:2:1), and homogenised with an IKA Ultra-Turrax disperser (IKA-Werke GmbH, Staufen, Germany) for 2 minutes. Phase separation was achieved by centrifugation, and the chloroform phase was transferred to new vessels with a Pasteur pipette. Afterwards, a known volume of chloroform (0.5–1 mL) was pipetted to pre-weighed tubes and evaporated overnight. The resulting dried residue was weighed and compared with the obtained DW to allow an accurate determination of the lipid fraction.

#### Biodiesel synthesis

Lipids were extracted directly from wet biomass as described by Yang *et al*.^56^ with modifications. Briefly, 100 g of wet microalgae paste were dispersed in 250 mL of absolute ethanol at reflux temperature for 120 minutes (EtOH-1). Afterwards, the crude ethanol extract was separated from the remaining biomass by centrifugation (4000 *g*, 10 minutes). The biomass was further extracted using the same aforementioned conditions for 60 (EtOH-2, 200 mL) and 30 (EtOH-3, 150 mL) minutes. All extracts were pooled and the ethanol was evaporated from the mixture using a rotatory evaporator.

Extracted lipids were converted to biodiesel by acid catalysed transesterification using the method described in Gangadhar *et al*.^10^ with modifications. Briefly, a solution of methanol and concentrated sulphuric acid (2% H_2_SO_4_ in methanol) was added to a round bottom flask containing the extracted lipids. The reaction mixture was stirred at reflux temperature for approximately 4 hours. The conversion of triacylglycerols into FAME was followed by thin-layer chromatography, using hexane and ethyl acetate (95:5 v/v) as mobile phase. Upon reaction completion, the solvent was evaporated using a rotatory evaporator and the fatty acids were sequentially extracted three times with hexane. The resulting fractions were pooled and washed with distilled water to neutralize the acid.

#### Fatty acid methyl esters profile

Produced biodiesel was analysed on a Bruker GC-MS (Bruker SCION 456/GC, SCION TQ MS) equipped with a ZB-5MS (30 m × 0.25 mm internal diameter, 0.25 μm film thickness, Phenomenex) capillary column using helium as carrier gas. The temperature program was 60 °C (1 min), 30 °C min^−1^ to 120 °C, 5 °C min^−1^ to 250 °C, and 20 °C min^−1^ to 300 °C (2 min). Injection temperature was 300 °C. For identification and quantification of the FAME total ion mode was used. Because of differences in the response factors, for each FAME separate calibration curves were determined in triplicate, using the Supelco^®^ 37 Component FAME Mix (Sigma-Aldrich, Sintra, Portugal) commercial standard. In the case where no standard was available, the response factor of the most similar FAME, in terms of structure, was used. Results are expressed as a percentage of total FAME content.

#### Biodiesel properties

The density of the produced biodiesel was determined at 15 °C using a certified Lenz pycnometer. Biodiesel kinematic viscosity was measured at 40 °C using a micro Ubbelohde viscometer in accordance with ISO 3105. Glycerol and acylglycerols contents were determined as per EN14105 method. Group I and II metals, as well as the phosphorous content, were determined by a microwave plasma atomic emission spectrometry (MP-AES 4200, Agilent technologies) according to Agilents’ technical note (5990-9005EN).

The CN of the FAME mix (CN_mix_) was estimated using the equation described in Knothe^57^, relating the CN (CN_c_) and relative amount (A_c_) of each FAME in the biodiesel mixture:


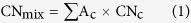


The oxidation stability was estimated using a Rancimat (model 743) according to the standard EN 14112:2003.

IV was calculated using the factors estimated for different FAME according to the EN14214.

The CFPP was calculated using the equations proposed by Ramos *et al*.^58^. This model relies on the estimation of the CFPP through the determination of the long chain saturated factor (LCSF) in accordance with the following equations:


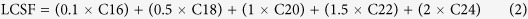



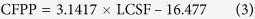


## Additional Information

**How to cite this article**: Pereira, H. *et al*. Isolation of a euryhaline microalgal strain, *Tetraselmis* sp. CTP4, as a robust feedstock for biodiesel production. *Sci. Rep.* **6**, 35663; doi: 10.1038/srep35663 (2016).

## Figures and Tables

**Table 1:** Biomass and lipid productivities previously reported for *Tetraselmis* species and in the present work.

Species	Biomass productivity (g L^−1^ d^−1^)	Lipid productivity (mg L^−1^ d^−1^)	Reference
*Tetraselmis* sp. (F&M-M34)	0.30	43.4	Rodolfi *et al*.^59^
*Tetraselmis suecica* (F&M-M33)	0.32	27.0	Rodolfi *et al*.^59^
*Tetraselmis suecica* (F&M-M35)	0.28	36.4	Rodolfi *et al*.^59^
*Tetraselmis* sp.	n.a.	22.7	Huerlimann *et al*.^31^
*Tetraselmis* sp.	n.a.	18.6	Huerlimann *et al*.^31^
*Tetraselmis* sp.	n.a.	22.2	Huerlimann *et al*.^31^
*Tetraselmis* sp. (MUR 167)	0.09†	25.8†	Fon-Sing & Borowitzka^60^
*Tetraselmis* sp. (MUR 219)	0.08†	30.3†	Fon-Sing & Borowitzka^60^
*Tetraselmis* sp. (MUR 230)	0.09†	43.2†	Fon-Sing & Borowitzka^60^
*Tetraselmis* sp. (MUR 231)	0.20†	85.5†	Fon-Sing & Borowitzka^60^
*Tetraselmis* sp. (MUR 232)	0.09†	25.8†	Fon-Sing & Borowitzka^60^
*Tetraselmis* sp. (MUR 233)	0.17†	58.0†	Fon-Sing & Borowitzka^60^
*Tetraselmis* sp. (CTP4; N+)	0.29	24.6	Present work
*Tetraselmis* sp. (CTP4; N−)	0.25	52.1	Present work

^†^Productivities were determined on an ash free dry weight basis.

**Table 2:** Fatty acid profile of the biodiesel synthesised from *Tetraselmis* sp. CTP4.

Fatty acid	Name	Biodiesel (%)
C16:0	Palmitic acid	24.13
∑*SFA*		*24.13*
C16:1	Palmitoleic acid	14.70
C18:1	Oleic acid	25.67
∑*MUFA*		*40.37*
C16:2	Hexadecadienoic acid	0.44
C18:2	Linoleic acid	23.17
C16:3	Hexadecatrienoic acid	9.89
C18:3	Linolenic acid	1.23
C20:5	Eicosapentaenoic acid	0.77
∑*PUFA*		*35.50*

**Table 3:** List of biodiesel properties analysed in the biodiesel produced from lipids of *Tetraselmis* sp. CTP4 and the limits established by each standard (EN 14214 and ASTM D6751).

Biodiesel properties	Unit	Biodiesel	EN 14214	ASTM D6751
FAME content	% (m/m)	96.72	≥96.50	—
Density (15 °C)	Kg L^−1^	0.85	0.86–0.90	—
Viscosity (40 °C)	mm^2^ s^−1^	3.64	3.50–5.00	1.90–6.00
Cetane number	—	51.33	≥51	≥47
Oxidation stability	hours	4.74	>6	>3
Iodine value	g I/100 g	110.63	≤120	—
Linolenic acid	% (m/m)	1.23	≤12.0	—
PUFA ≥4 db	% (m/m)	0.77	≤1.00	—
Monoglyceride content	% (m/m)	<0.1	≤0.80	—
Diglyceride content	% (m/m)	<0.05	≤0.20	—
Triglyceride content	% (m/m)	<0.05	≤0.20	—
Free glycerol	% (m/m)	<0.001	≤0.02	≤0.02
Total glycerol	% (m/m)	<0.05	≤0.25	≤0.24
Group I metals (Na + K)	mg kg^−1^	0.45	≤5.0	≤5.0
Group II metals (Ca + Mg)	mg kg^−1^	0.05	≤5.0	≤5.0
Phosphorus content	mg kg^−1^	24.02	≤4.0	≤10.0
CFPP	°C			
Summer		−8.89	≤ −5/+5*	
Winter			≤−5/−20*	

^*^Country-dependent (values not included in EN 14214); limits describe the range of maximum values allowed by the legislation applied in Austria, France, Germany, Greece, Ireland, Italy, Netherlands, Portugal, Spain and United Kingdom. n.d.: not detected.
